# A Translational In Vivo and In Vitro Metabolomic Study Reveals Altered Metabolic Pathways in Red Blood Cells of Type 2 Diabetes

**DOI:** 10.3390/jcm9061619

**Published:** 2020-05-27

**Authors:** Martina Palomino-Schätzlein, Rubén Lamas-Domingo, Andreea Ciudin, Patricia Gutiérrez-Carcedo, Rosó Marés, Carolina Aparicio-Gómez, Cristina Hernández, Rafael Simó, José Raúl Herance

**Affiliations:** 1 NMR Facility, Centro de Investigación Príncipe Felipe, C/Eduardo Primo Yúfera 3, 46012 Valencia, Spain; rlamas@cipf.es; 2 Diabetes and Metabolism Research Unit, Vall d’Hebron Research Institute, Autonomous University of Barcelona, Passeig de la Vall d’Hebron 119-129, 08035 Barcelona, Spain; aciudin@vhebron.net (A.C.); biotecpat@gmail.com (P.G.-C.); cristina.hernandez@vhir.org (C.H.); rafael.simo@vhir.org (R.S.); 3 CIBERDEM (ISCIII), Av. Monforte de Lemos, 3-5, 28029 Madrid, Spain; 4 Medical Molecular Imaging Research Group, Vall d’Hebron Research Institute, CIBBIM-Nanomedicine, and Autonomous University of Barcelona, Passeig de la Vall d’Hebron 119-129, 08035 Barcelona, Spain; rmares@clinic.cat (R.M.); carolina.aparicio@vhir.org (C.A.-G.); 5 CIBER-bbn (ISCIII), Av. Monforte de Lemos, 3-5, 28029 Madrid, Spain

**Keywords:** erythrocytes, type 2 diabetes mellitus, NMR, metabolomics, biomarker

## Abstract

Clinical parameters used in type 2 diabetes mellitus (T2D) diagnosis and monitoring such as glycosylated haemoglobin (HbA1c) are often unable to capture important information related to diabetic control and chronic complications. In order to search for additional biomarkers, we performed a pilot study comparing T2D patients with healthy controls matched by age, gender, and weight. By using ^1^H-nuclear magnetic resonance (NMR) based metabolomics profiling of red blood cells (RBCs), we found that the metabolic signature of RBCs in T2D subjects differed significantly from non-diabetic controls. Affected metabolites included glutathione, 2,3-bisphophoglycerate, inosinic acid, lactate, 6-phosphogluconate, creatine and adenosine triphosphate (ATP) and several amino acids such as leucine, glycine, alanine, lysine, aspartate, phenylalanine and tyrosine. These results were validated by an independent cohort of T2D and control patients. An analysis of the pathways in which these metabolites were involved showed that energetic and redox metabolism in RBCs were altered in T2D, as well as metabolites transported by RBCs. Taken together, our results revealed that the metabolic profile of RBCs can discriminate healthy controls from T2D patients. Further research is needed to determine whether metabolic fingerprint in RBC could be useful to complement the information obtained from HbA1c and glycemic variability as well as its potential role in the diabetes management.

## 1. Introduction

Type 2 diabetes mellitus (T2D) comprises 90% of patients with diabetes and is caused by a combination of genetic and lifestyle factors, including insulin resistance, excess body weight and physical inactivity [1,2]. Currently, there are about 463 million of T2D patients worldwide and the disease causes 4.2 million of deaths annually. These figures, could be doubled in 2030 [3,4]. Thus, this disease could be considered as the worldwide pandemic of the 21st century, causing both severe health problems and an increase of the healthcare costs [5,6,7].

HbA1c is used by physicians to obtain an overall picture of average blood glucose levels over a long time period (around 2 months) [8,9,10,11]. Patients with higher HbA1c values are associated with a greater risk of developing diabetes-related complications. However, HbA1c has a low rate for predicting diabetes-related chronic complications, and does not capture glucose variability and other metabolic changes unrelated to glycaemia itself [12,13,14]. This makes the exploration of new biomarkers an attractive field to improve the management of diabetes. In this regard, new diagnostic approaches for precision medicine could complement or even improve the current parameters used in the clinic such as glycosylated haemoglobin (HbA1c) or blood glucose levels [15,16,17]. For instance, additional biomarkers could help to select a more appropriate treatment, or provide a better classification in terms of risk of comorbidities.

RBCs are rather unique body cells, since they have lost all organelles when mature and they only conserve a few metabolic pathways for obtaining energy and reducing the power consumption for the key functions they need to fulfil [18,19]. This makes RBCs highly sensitive to any disorder [20]. In addition, RBCs are involved in the transport and delivery of nutrients such as amino acids. It has been shown that the metabolism of RBCs is altered in T2D, including the glycosylation of the heme group by an excess of glucose [21,22,23,24,25]. This heme glycosylation tends to shift the oxygen dissociation curve to left, leading to an increase in haemoglobin–oxygen affinity and reduced oxygen delivery to tissues [26,27,28]. Furthermore, metabolite transport and basal metabolism of RBCs can be compromised in T2D, thus impairing the delivery of certain compounds to several tissues [29,30,31].

In this context, the study of the metabolic profile of RBCs is a suitable approach for personalized medicine in T2D. We recently set up a new protocol [32] that allows the quantification of more than 80 different metabolites in RBCs by ^1^H nuclear magnetic resonance (NMR) that can be linked to different metabolic pathways. Although several studies about the metabolomic profile of serum/plasma samples have been published [33,34,35], little information exist regarding NMR-based studies about RBCs, and to the best of our knowledge there are no studies focused on the metabolomic assessment of RBCs in T2D. RBCs are easily available and may provide valuable additional or complementary information in T2D. For instance, the current biomarker for T2D, Hb1Ac, is measured in RBCs and not in plasma. This suggests that the RBC is especially sensitive to T2D and, therefore, thus could eventually provide additional biomarkers [36]. In addition, the metabolites identified in RBCs are different to the metabolites than can be quantified from plasma and several metabolites that can only be found at low concentrations in plasma are much more concentrated in RBCs (i.e., nicotinamide adenine dinucleotide derivatives, glutathione) [32]. Moreover, cells may be less sensitive to on-off changes than plasma/serum samples, reducing false positive/negative results [37]. Furthermore, metabolomics profiling of RBCs can improve the study on the effect of hypoxia in T2D since these cell act as oxygen delivering systems [38,39,40]. Finally, RBCs represent approximately the 70% of the total cell body counting [41], thus it is important to know more about how their metabolism is affected by T2D. 

On this basis, we have performed a first proof-of-concept study aimed at examining the differences in the metabolic profile of RBCs between patients with T2D and healthy controls. This approach will allow us to generate a new approach for the management of this complex disease. For this purpose, a case-control study was designed with T2D patients and healthy control individuals, which was further validated with an independent cohort. The metabolomic profile of RBCs isolated from peripheral blood samples was compared between cohorts to find metabolites and the metabolic pathways affected by T2D. In a complementary study, the initial findings were reproduced in vitro by incubating RBCs with different glucose concentrations to mimic T2D [42]. 

## 2. Materials and Methods

### 2.1. Solvents and Reagents

Unless otherwise indicated, the solvents and reagents employed were purchased from Sigma-Aldrich (Madrid, Spain), Gibco (Madrid, Spain), Thermo Fisher Scientific (Waltham, MA, USA), Biowest (Nuaillé, France) Falcon BD (Madrid, Spain) or Eurisotop (Gif sur Yvette, France) and were used in the form in which they were supplied. Gases were supplied by Air-Liquide (Valencia, Spain).

### 2.2. Ethics Statement

The study was conducted according to the tenets of the Helsinki Declaration. The Ethic Committee of the Vall d’Hebron University Hospital approved all procedures (protocol number PR(AG)234/2015). Written informed consent was obtained from all the participants before any action. 

### 2.3. Subjects

Initial cohorts of 22 T2D patients and 21 healthy individuals were recruited from February to June 2018 at the Outpatient’s Department of the Endocrinology Service of Vall d’Hebron University Hospital. Furthermore, two independent cohorts of 12 T2D patients and 6 healthy controls were recruited after several months in the same hospital from January to May 2019. All cohorts were matched for age, gender and body mass index (BMI). In addition, 4 controls (2 age-matched women and men) were recruited at the Outpatient’s Department of the Endocrinology Service for the in vitro study. Smokers, alcohol drinkers and subjects with previous cardiovascular events or other comorbidities were excluded from the study. An overview of the characteristic of T2D patients and controls is shown in Table 1.

### 2.4. Peripheral Blood Samples

Peripheral blood was collected under fasting conditions, stored at 4 °C and processed within the first hour. Anthropometrical parameters of weight, waist and height were measured just before collecting the blood samples. Biochemical analysis was performed at the biochemistry core facilities of Vall d’Hebron University Hospital. Plasmatic concentrations of glucose, HbA1c, high-density lipoprotein (HDL), low-density lipoprotein (LDL), triglycerides (TG) and insulin were measured in all the subjects. The BMI and the homeostasis model assessment of insulin resistance (HOMA-IR) were determined by the standard equations using anthropometrical and biochemical data of the subjects. [43,44] The HbA1c of the in vitro study was determined by the classical enzymatic method at the biochemistry core facilities of Vall d’Hebron University Hospital [45].

### 2.5. Isolation and Storage of RBCs from Peripheral Blood

A pellet of RBCs was obtained from 5 mL of freshly extracted peripheral blood which was poured into a quartz tube with 10 mL of Ficoll and left standing around 30 min until 2 phases were separated by gravity. Then, the supernatant was poured off and the pellet was washed twice with 10 mL of cool Hank’s balanced salt solution in a centrifuge at 200× *g* and 4 °C for 10 min without brakes. Cell counting was performed using a dilution 1:1000 of cells on a Neubauer chamber and the purity was tested with flow cytometry. For storage, 200 µL of ice-cold methanol for quenching was added per 10 million cells and the samples frozen directly at −80 °C. 

For in vitro studies, washed RBCs were resuspended in 10 mL of non-supplemented Roswell Park Memorial Institute medium (RPMI) to obtain a total volume of 12 mL approximately. Total of 10 μL of this solution was diluted with 990 μL of medium to count cells in a Neubauer chamber and later the volume was adjusted to have around 200 M of RBC cells/mL.

### 2.6. Incubation of RBCs at Different Glucose Concentration and Times 

RBCs were incubated with different amounts of glucose, simulating hyperglycaemia conditions. This methodology has been used previously by some authors to assess membrane transport [46] and the glucose-6-phosphate dehydrogenase deficiency [47] in T2D. For the experiments, the previous mixture of RBCs in non-supplemented RPMI, were supplemented with 240, 570 and 1200 μL of glucose 20% in water to obtain a glucose concentration of 4.5, 9, 20 mg/mL respectively. The mixture was aliquoted in 1 mL triplicates for 0 and 24 h, leaving this last sample in an incubator at 37 °C at different glucose concentrations. Then, 500 μL of the mixture was used directly for HbA1c analysis. The other 500 μL were centrifuged at 200× *g* and 4 °C for 2 min, the supernatant was discarded and the pellet was washed twice with 1 mL of cool Hank’s balanced salt solution in the centrifuge at 200× *g* and 4 °C for 2 min. Finally, 1600 μL of cold methanol was added and the samples were placed at −80 °C until the metabolomic analysis was carried out.

### 2.7. HbA1c Quantification of RBCs Incubation Samples

To set up a suitable method to quantify HbA1c in cultured RBCs, several amounts of RBCs isolated from 10 mL of peripheral blood (the whole RBC pellet, 200,000, 100,000, 60,000, and 20,000 cells) were cultured in RPMI, and HbA1c was quantified after 24 h. The results obtained for HbA1c were compared with the quantification performed in the same amount of whole blood. The assay (five replicates) proved that only the whole RBCs pellet resuspended in the same volume of complete peripheral blood provided a real and constant HbA1c value for these cells, and this protocol was used to perform the in vitro RBCs experiments. 

Samples were analysed in the biochemistry core facilities at the Vall d’Hebron University Hospital using the clinical routine standard methodologies [45]. Previously, to set-up and validate the quantification of HbA1c in cultured RBCs, several conditions were assessed to obtain a proper HbA1c value by taking into account the concentration of cells, preparation of samples as well as the media volume. 

### 2.8. Extraction of Polar Metabolites

Frozen samples were placed on ice and allowed to thaw for 5 min. Total of 800 µL of chloroform at 4 °C was added. After 10 min, the samples were homogenized with a vortex, resuspended with a pipette and transferred to a plastic tube. For uniform cell breakage, the samples were placed in liquid nitrogen for 1 min and then allowed to thaw on ice for 2 min. This step was repeated twice. Afterwards, 1250 µL of distilled water and 1250 µL of chloroform were added and the sample was vortexed. Then, samples were centrifuged at 13,000× *g* for 20 min at 4 °C to separate the phases. The upper phase that contains polar metabolites in a mixture of water/methanol was separated from the interphase and the lower chloroform phase and then lyophilised for 2 h. Extracts were stored at −80 °C until preparing samples for NMR analysis.

Frozen samples for NMR analysis were placed on ice and allowed to thaw for 5 min. Total of 550 µL of NMR buffer (100 mM Na_2_HPO_4_ in D_2_O at pH 7.4) containing 0.1 mM deuterated trimethylsilylpropanoic acid as internal standard was added to the samples and the mixture was transferred to a 5-mm NMR tube. Samples were analysed the same day and stored at 4 °C for 15 min before analysis.

### 2.9. NMR Experiments

NMR spectra were recorded at 27 °C on a Bruker AVII-600 spectrometer using a 5 mm triple resonance cryoprobe and processed using Topspin3.2 software (Bruker Biospin; Billerica, MA, USA). ^1^H 1D noesy NMR spectra were acquired with 256 free induction decays (FIDs). 64 k data points were digitalised over a spectral width of 30 ppm for an optimal baseline correction. A 4s relaxation delay was incorporated between FIDs and water presaturation was applied for aqueous samples. The FID values were multiplied by an exponential function with a 0.5 Hz line broadening factor. A water presaturation pulse of 25 Hz was applied throughout the relaxation delays to improve solvent suppression.

Total correlation spectroscopy (TOCSY) and multiplicity heteronuclear single quantum correlation (HSQC) were acquired for representative samples. For each of these experiments, 256–512 t1 increments were used and 32–96 FIDs were collected. The relaxation delay was set to 1.5 s and the experiments were acquired in the phase-sensitive mode. TOCSY spectra were recorded using a standard MLEV-17 pulse sequence with mixing times (spin-lock) of 65 ms.

### 2.10. Data Analysis

^1^H-NMR signals from the spectra were assigned to the metabolites with the help of 2D experiments, spectral databases Human Metabolome Database (HMBD)^49^ and the Biological Magnetic Resonance Bank (BMRB)^50^ [48,49]. In ambiguous cases, the assignment was confirmed by spiking the spectra with reference compounds. Spectra were normalised to total intensity, excluding glucose and solvent signals, to minimise the differences in concentration and experimental error during the extraction process. Optimal integration regions were defined for each metabolite, an attempt being made to select the signals without overlapping (Supplementary Table S1). Integration was performed with global spectrum deconvolution (GSD) in MestreNova 12 (Mestrelab Research S. L.).

For multivariate analyses, metabolite tables generated from spectra integration were univariate scaled (each value being divided by the standard deviation of each variable) or pareto scaled (each value being divided by the square root of the standard deviation of each variable), and mean centred for an easier interpretation of the data and to take the variation of small signals into account. Principal component analysis (PCA), projection on latent structure (PLS) and orthogonal projection on latent structure discriminant analysis (OPLS-DA) were performed with SIMCA-P 14.0 (Umetrics; Umeå, Sweden). OPLS-DA and PLS models were described with R2Y(cum) (representing the cumulative SS of all the y-variables explained by the extracted components) and Q2(cum) (describing the cumulative Q2 for all y-variables for the extracted components). Four and five samples had to be excluded for PLS analysis versus glucose and HbA1c, respectively, as no updated clinical glucose and HbA1c data were available for the analysis of metabolites that were relevant in the OPLS-DA model; score plots (representing scores related to prediction Y t [1] versus scores related to the first orthogonal component t0[1]) in combination with S-plots (plotting the modelled covariation (*p* [1]) versus the modelled correlation (p(corr)) were analysed [50]. The glucose signal, due to its high intensity and correlation to plasmatic glucose, was excluded from OPLS-DA analysis. For cross validation, a previously reported approach was used [51]. Briefly, in two sub rounds, data were first kept out observation-wise (row-wise) to get a set of loading vectors, and second data were kept out variable-wise (column-wise) to get a set of score vectors. OPLS-DA and PLS models were validated in SIMCA-P by 100 times permutation (where the overfit of the model is measured by the intercept of the regression line of the correlation coefficient between the original y-variable and the permuted y-variable on the *x*-axis versus the cumulative R2 and Q2 on the *Y*-axis) and analysis of variance testing of cross-validated predictive residuals (CV-Anova) [52]. CV-Anova is a significance test for the Q2YCV cross validation using the F-distribution, it is based on an ANOVA assessment of the cross-validatory (CV) predictive residuals of the model. 

For the correlation of metabolite concentrations with biochemical and anthropometrical parameters, a Spearman’s rank correlation coefficient analysis was performed using R commander (The R Foundation).

For the biological interpretation of the results and the identification of metabolic pathways the Kegg Data Base and MetPA (Metaboanalyst) were used [53,54].

Data plots, Shapiro-Wilk normality test and *t*-tests were performed with normalised concentration data in R (The R Foundation) and Graphpad Prism 8 [55].

## 3. Results

### 3.1. General Characteristics of Cohorts in Study

A clinical trial was performed with 22 T2D patients and 21 controls (CT) in order to correlate the metabolic profile of their RBCs with T2D and several biochemical and anthropometrical parameters. Subsequently, a second independent group of 12 patients and 6 control individuals was analysed to validate the results (CT and T2D validation). The main biochemical and anthropometrical clinical features of cohorts are shown in Table 1. As expected, HB1Ac levels were significantly higher in T2D groups than in control subjects. 

### 3.2. Altered Metabolic Profile of RBCs in T2D Patients 

**Figure 1:**
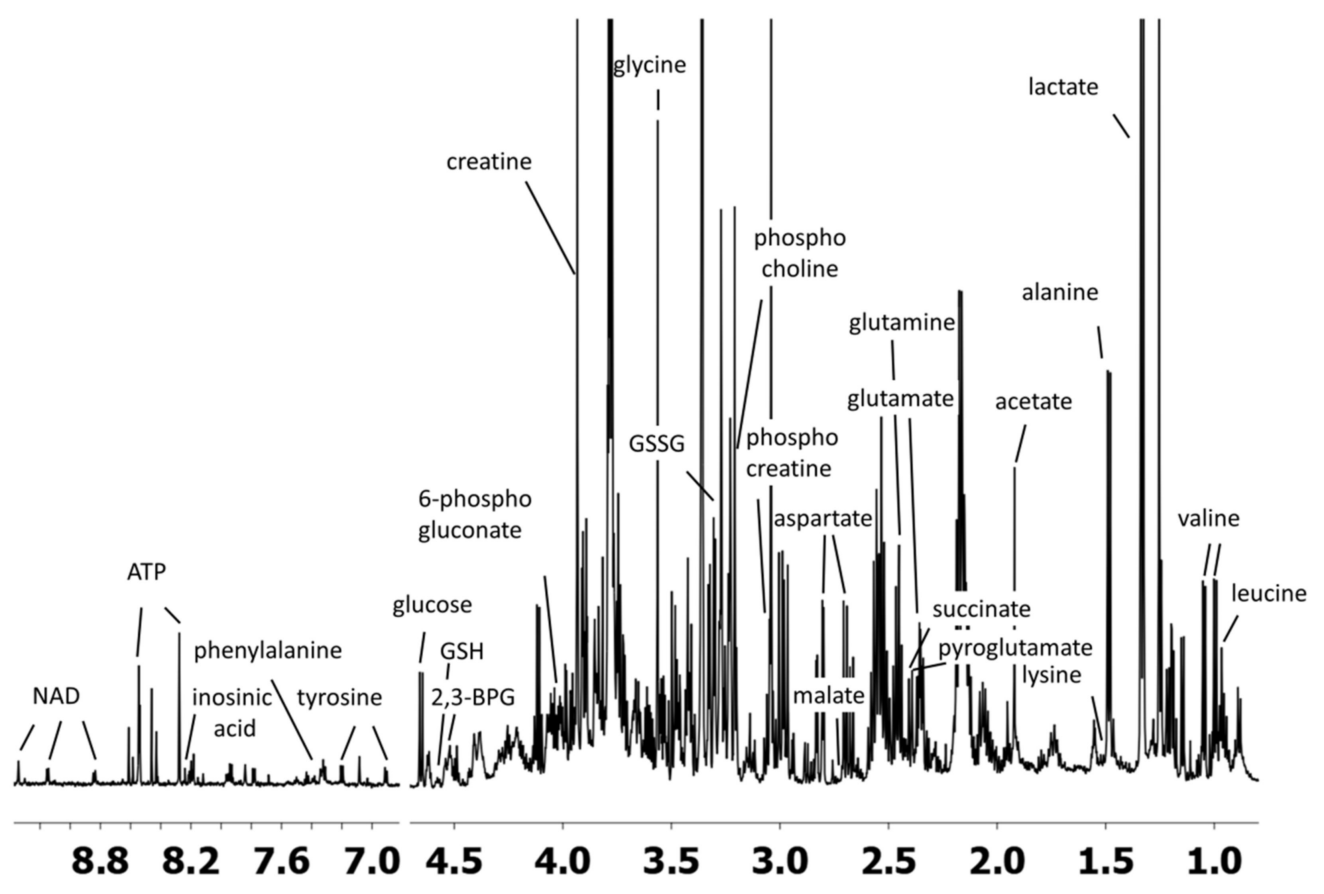
Representative ^1^H NMR spectrum of the polar metabolic extract of RBCs. Determination of polar metabolites in the ^1^H-NMR spectra acquired at 600 MHz and 27 °C with 256 free induction decays (FID). NAD = nicotinamide adenine dinucleotide, ATP = adenosine triphosphate, GSH = reduced glutathione, GSSG = oxidized glutathione, 2,3-BPG = 2,3-Bisphosphoglyceric acid.

A representative spectrum of RBC extracts is shown in Figure 1, where several metabolites could be identified and quantified, based on previous data [56,57] and 2D-NMR spectra (Supplementary Table S1). 

**Figure 2:**
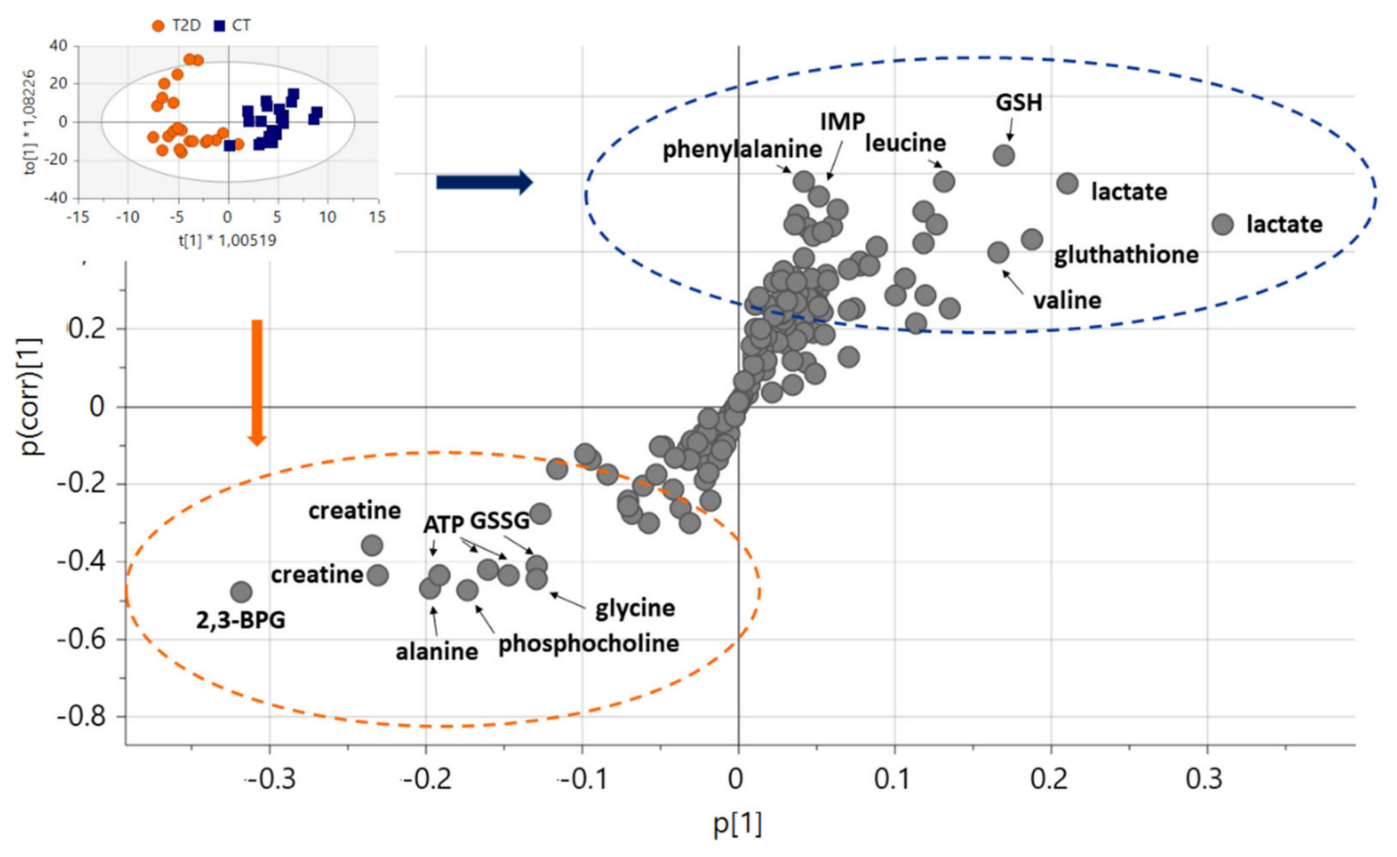
OPLS-DA analysis of the metabolomic profile of RBCs of control (CT) and Type 2 Diabetes Mellitus (T2D) patients. R2Y(cum) = 0.817, Q2(cum) = 0.624. Permutation: R2 = (0.000, 0.312), Q2 = (0.000, −0.494). Analysis of variance testing of cross-validated predictive residuals (CV-Anova): *p*-value = 4.2 × 10^5^. IMP = inosine monophosphate, ATP = adenosine triphosphate, GSH, = reduced glutathione, GSSG = oxidized glutathione, 2,3-BPG = 2,3-bisphosphoglyceric acid.

The resulting metabolomic profiles of the cohorts were submitted to PCA analysis, confirming that no significant outliers were present (Supplementary Figure S1). In order to analyse the differences between CT and T2D patients, an OPLS-DA model was built, which proved an excellent discrimination between these two cohorts (Figure 2). The S-plot that is depicted in the figures shows the key metabolites that differed between groups. 

In order to test the robustness of this model, we applied it to predict the class of the 12 samples from the T2D validation group and the 6 samples from the CT validation group, obtaining a correct prediction for 10 T2D patients and 5 control individuals (Table 2). A final OPLS-DA model of all samples could be obtained (Supplementary Figure S2), which was very similar to the initial OPLS-DA model (Figure 2) and had excellent validation values. 

**Figure 3:**
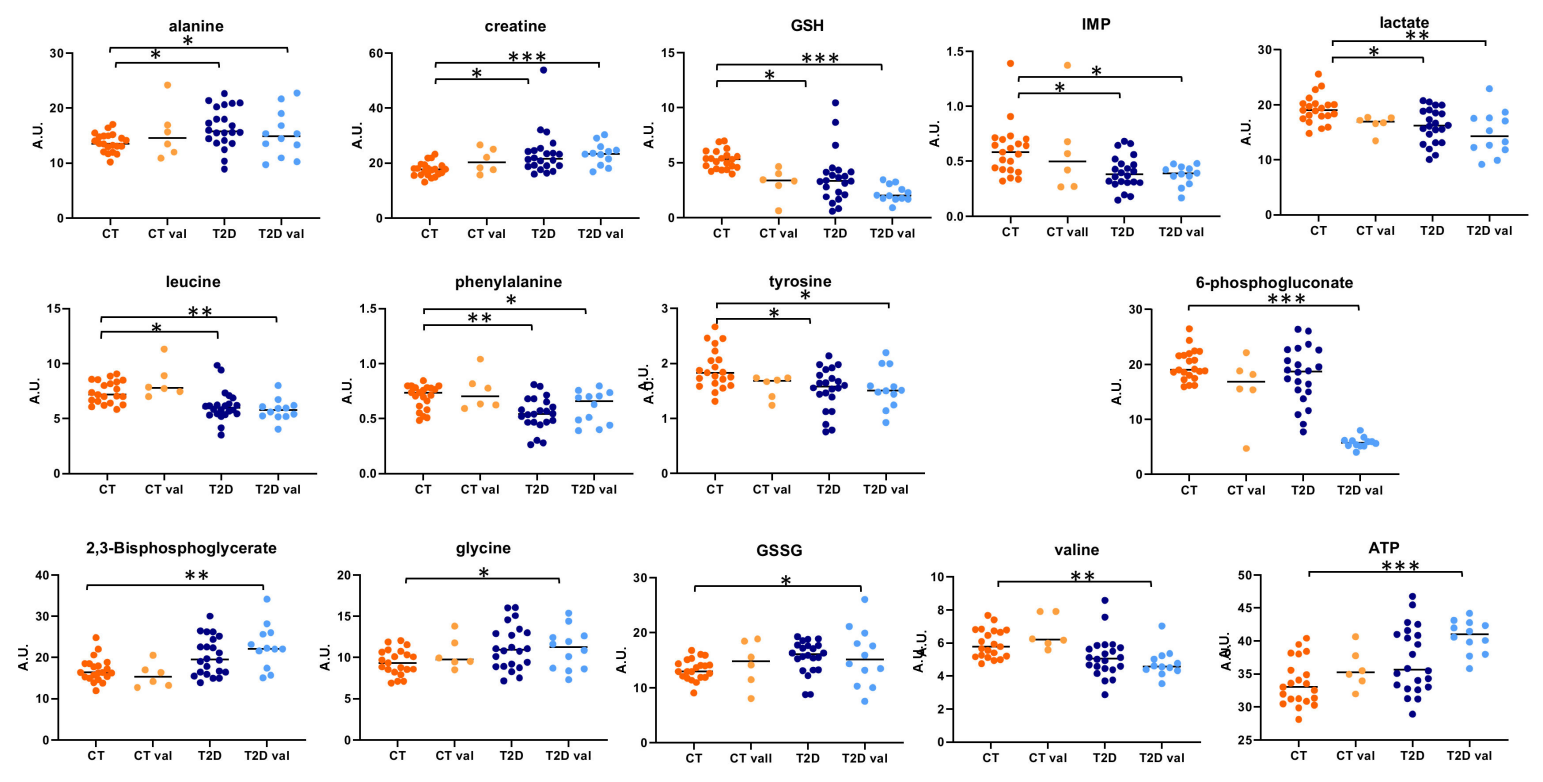
Representation of elected metabolites that change significantly between diabetic patients and healthy controls. Relative normalised intensities are represented. ATP = adenosine triphosphate, GSH, = reduced glutathione, GSSG = oxidized glutathione. Statistical significance was determined using the Holm-Sidak method, with alpha = 0.05. Each row was analysed individually, without assuming a consistent standard deviation (SD). * = *p*-value < 0.05, ** = *p*-value < 0.01, *** = *p*-value < 0.001. *p*-values between the CT and the T2D group, and the CT and the T2D validation group are indicated.

In order to further define the metabolites that differed between the CT and the T2D cohorts, the metabolomics data were analysed using univariate analysis. Relevant results are summarised in Supplementary Table S2 and in Figure 3. As can be seen, a series of metabolites followed a similar behaviour in the T2D and T2D validation group, when compared with the CT group. For instance, GSH, IMP and lactate were decreased while creatine was increased. Alanine was incremented while other amino acids (tyrosine, leucine and phenylalanine) were decremented. In the case of 2,3-bisphophoglycerate (2,3-BPG), glycine, GSSG and ATP (all increased in T2D validation) and valine (decreased in T2D validation), changes between the CT and the T2D group were not statistically significant, but followed a similar tendency. Interestingly, the levels of 6-phosphogluconate decreased significantly in the T2D validation group but not in the T2D group. 

**Figure 4:**
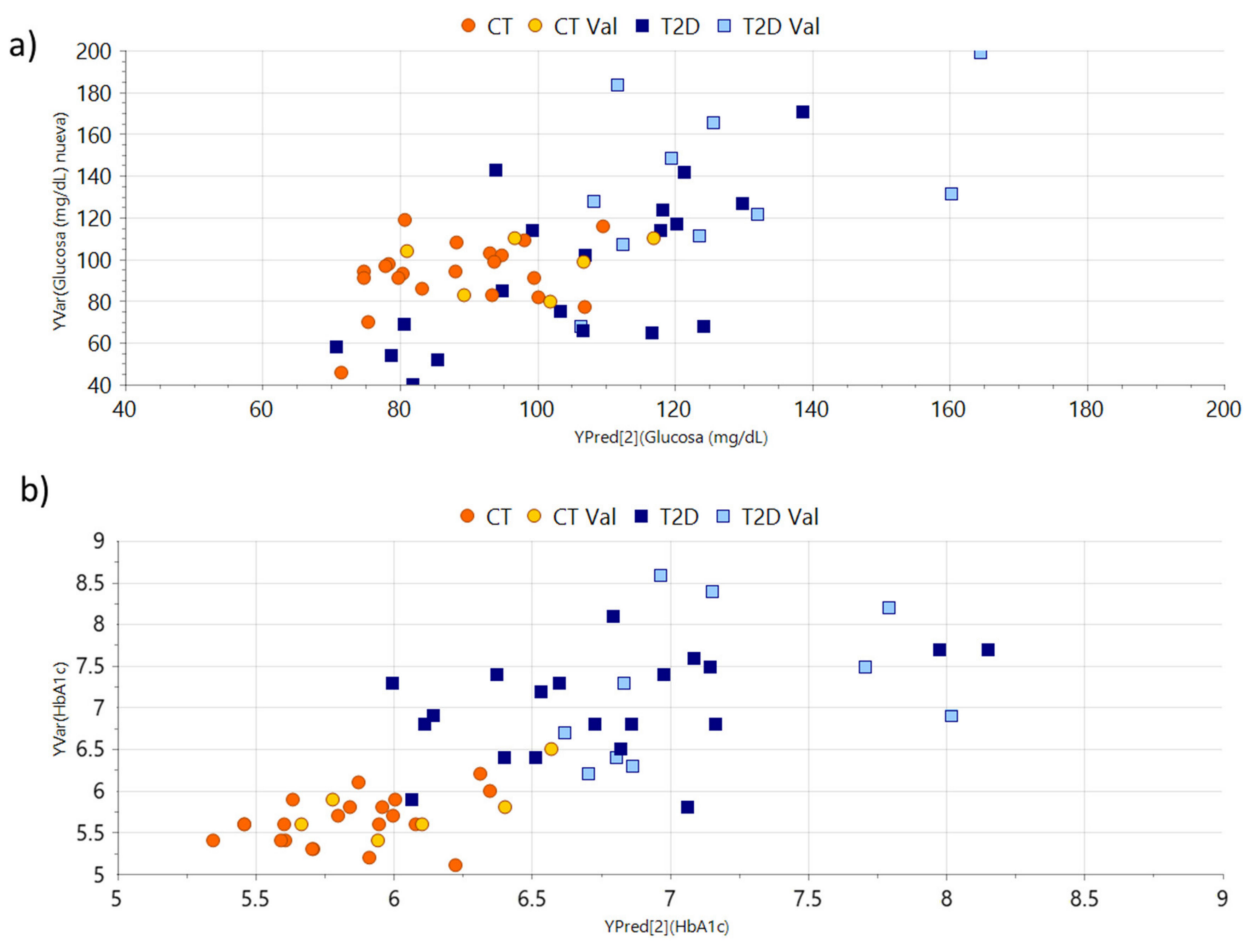
Partial least squares (PLS) discriminant analysis observed versus predicted plot for glucose and glycosylated haemoglobin (HbA1c). (**a**) PLS glucose observed versus predicted plot of a two-component model: R2Y (cum) = 0.406 Q2 (cum) = 0.188. Permutation: R2 = (0.0, 0.177), Q2 = (0.0, −0.169). CV-Anova: *p*-value = 0.018. (**b**) PLS HbA1c observed versus predicted plot of a two-component model: R2Y (cum) = 0.573, Q2 (cum) = 0.411. Permutation: R2 = (0.0, 0.146), Q2 = (0.0, −0.181). CV-Anova: *p*-value = 7.6 × 10^−7^). Data were pareto scaled and mean centred.

To complete the prediction between the metabolic profile of RBCs and the biochemical and anthropometrical parameters of patients, PLS analysis of metabolites versus biochemical and anthropometrical data altered between cohorts was performed. PLS correlations could be obtained for HbA1c and plasmatic glucose, as reflected in Figure 4. Metabolites that contributed significantly to the models are represented in Table 3 and Table 4. The variable importance for projection (VIP) values reflect the importance of the variables to the model. VIP values higher than 1 point to variables with large importance. The loading coefficients indicated how much the variables participate in the modelling of y. They can be positive or negative, providing information about the sense of the correlation (proportional or anti-proportional). 

Several metabolites proved to have a similar correlation to plasmatic glucose as to HbA1c, including 2,3-BPG, glucose, lactate and glutamine. However, other compounds had a higher significance for one of the models, such as creatine, alanine, ATP and GSH in the case of HbA1c, and glucose, 6-phosphogluconate, GSSG, pyroglutamate and aspartate in the case of plasmatic glucose. 

To further complete our analysis, non-parametric Spearman’s correlations were made between selected metabolites and either plasma glucose levels or HbA1c (Table 5). This approach allowed us to obtain individual correlations providing more robustness to the results. Metabolites were classified in two groups according to their correlation p value: correlated (*p* ≤ 0.05); non-correlated (*p* > 0.05). Our analysis revealed that creatine, GSH, 2,3-BPG, 6-phosphogluconate, glucose, glycine, ATP, aspartate, inosinic acid, leucine, lysine, phenylalanine, tyrosine, and valine were correlated to HbA1c. Spearman correlation confirmed the specific correlation of 2,3-bisphosphoglycerate, ATP, creatine, glycine, phenylalanine, tyrosine with HbA1c. In addition, lysine, 6-phosphogluconate, aspartate, glucose, GSH, inosinic acid, leucine and valine were correlated to plasmatic glucose levels. 

### 3.3. Altered Metabolic Profile of an In Vitro Model of T2D in RBCs 

In order to reproduce the metabolic changes induced by T2D in RBCs in vitro, we incubated RBCs with different glucose concentrations, to mimic the effects of glycaemia. Quantified HbA1c levels from triplicate experiments are shown in Table 6. As can be observed, HbA1c levels increased significantly after 24 h of incubation with glucose, and most significantly for cells treated with 20.0 g/L glucose.

The general metabolic profile of RBCs directly isolated from patients in vivo and after being cultured in RPMI in vitro was very similar and the same number of metabolites could be quantified. In order to compare the metabolic profile of RBCs in the in vitro and in vivo conditions, we selected the doses of 4.5 and 20 g/L to simulate the effects of increasing plasmatic glucose normally observed in patients with hyperglycaemia [42]. Metabolomics data of both conditions were compared (Table 7). As expected, not all changes could be reproduced, as the conditions of a cultured cell are very different from a cell being part of the biofluid in a complex organism. However, several of the metabolomic changes observed in T2D patients could be reproduced by the in vitro model, including the increase of glucose, succinate and glutamate, and the decrease in lactate and 6-phosphogluconate. On the other hand, changes of lysine, glutamine, pyroglutamate, valine, ATP and alanine levels had opposite tendencies in vivo than in vitro. Furthermore, several changes that were not observed in patients, were found in vitro, such as an increase in the organic acids acetate, malate and phosphocholine. 

## 4. Discussion 

Improved diagnostic approaches to combat T2D based on an early detection of patients more prone to develop comorbidities is an unmet need. HbA1c, the current parameter used in clinics with this purpose, does not achieve these goals due of different reasons. For example, HbA1c reflects the mean of blood glucose levels for long periods but does not take into account glycemic variability, the frequency and severity of hypoglycaemias or the presence of other diabetes-induced metabolites with deleterious effects. In addition, HbA1c has a low capacity to predict diabetic complications [12,13,14]. 

**Scheme 1:**
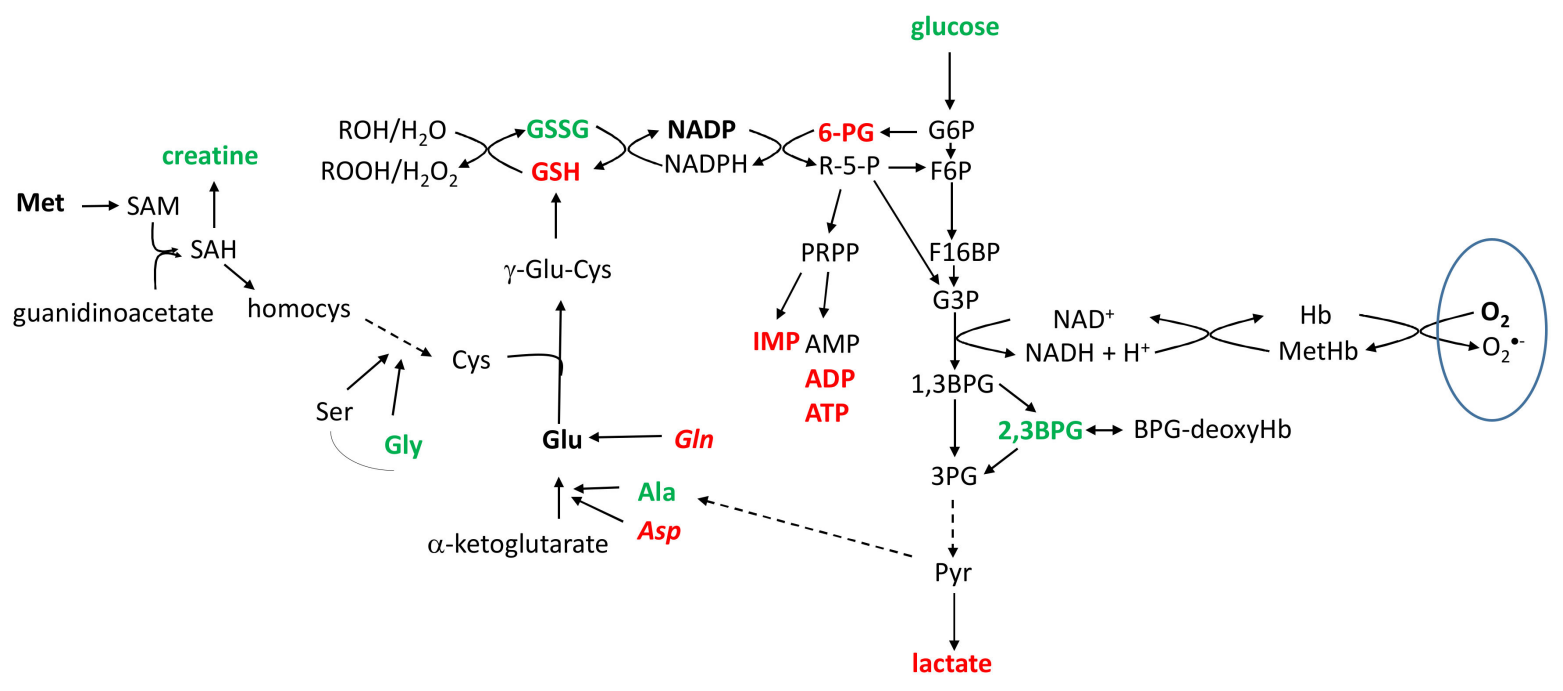
Main metabolic pathways of RBCs. Detected metabolites are labelled in bold. Metabolites that increase in T2D are labelled in green while those metabolites that are decreased are shown in red.

In this work, we provide a first evidence, showing that the metabolomic profile of RBCs in T2D patients is altered (Figure 3 and Supplementary Table S3). As expected the metabolic signature revealed an alteration of the several metabolic pathways (Scheme 1), thus offering an interesting starting point for biomarker research in T2D based on RBCs. 

GSH is one of the main endogenous antioxidant produced inside cells, which is able to modulate ROS levels acting as an scavenger of ROS, and therefore, governs the cellular oxidative stress response [58]. The reduction of GSH in RBCs in T2D has been previously observed [59] and it was linked to insulin resistance [60]. Whillier et al. [61] showed that the capacity to synthesize GSH was normal in RBCs of patients with T2D and attributed the decrease of GSH to the increase of oxidative stress produced by the elevated generation of reactive oxygen species (ROS) that occurs in T2D. Therefore, it seems that more GSH is converted into GSSG, which is increased in RBCs. This finding agrees with the increased GSSG plasma levels reported in T2D [62,63,64]. On the other hand, alanine, and glycine, which are required for the initial steps of GSH synthesis were found elevated in RBCs of T2D patients. This could indicate a compensation mechanism due to the loss of GSH. In the same line, the levels of creatine, related to cysteine synthesis, a key metabolite for GSH generation, were also incremented [65,66]. 

It has been previously described that oxidative stress activates the PPP pathway [67,68,69], to increase the production of reduced nicotinamide adenine dinucleotide phosphate (NADPH), required to reduce GSSG to GSH. Nevertheless, an increase of NADPH was not detected in our study, which could be related to the fact that the concentration of this metabolite is relatively low and close to the detection limit of NMR. However, the decrease in 6-phosphogluconate could indicate a high consumption of this metabolite to generate NADPH. Moreover, a 6-phosphogluconate dehydrogenase deficit is usually present in oxidative stress conditions similar to T2D [70,71]. Reduced 6-phosphogluconate could result in lower levels of phosphoribosyl pyrophosphate (PRPP), which could explain a reduced production of IMP. 

2,3-BPG is a modulator of haemoglobin oxygen affinity that plays an important role in blood oxygen transport and delivery. 2,3-BPG binds selectively to deoxyhemoglobin, enhancing the release of oxygen from the haemoglobin to adjacent tissues. 2,3-BPG is part of a feedback loop that can help to prevent tissue hypoxia in conditions where it is most likely to occur [72]. Increased erythrocyte 2,3-bisphosphoglycerate concentrations, as shown for T2D in our study, have been associated with increased haemoglobin glycosylation in patients with Type 1 diabetes [73]. Also, increased levels of 2,3-BPG in RBCs have been previously shown to play an important role in the transport and delivery of O_2_ in patients withT2D [74,75]. 

It is widely recognised that the flux through glycolysis slows down during diabetes [76]. Thus, the reduced lactate levels that we have observed seems to prove that glycolysis was also reduced in RBCs of T2D patients. In concordance, glucose catabolism through the PPP pathway was increased. In fact, it is well-known that a deficit of glucose-6-phosphate dehydrogenase exit in RBCs of patients with diabetes, thus leading to the reduction of the glycolysis metabolites in these cell.

Finally, the levels of several amino acids such as leucine, tyrosine, phenylalanine and lysine that are not synthesized in RBCs but transported by them, were also altered. Basically, it indicates that the load of amino acids in RBCs was reduced in T2D as opposed to molecules implicated in energetic and antioxidants pathways. Previous studies showed that the circulating plasmatic levels of most of these amino acids are increased in T2D [77]. 

In summary, we have identified a series of metabolites that are altered in the RBCs of DM2 patients and could be used as a fingerprint for this disease to manage DM2.

The metabolomic changes could be potential indicators of DM2 comorbidities. For instance, increased 2,3-BPG levels have been associated with hyperthyroidism and problems of O_2_ transport and delivery by RBCs in DM2 [74,75,78] while IMP could be related with the risk of haemolysis and membrane hyperpolarisation of RBCs, as well as haemoglobin deoxygenation [79,80,81,82]. GSH levels could also be related to ROS present in cardiovascular complications. On the other hand, the amino acid leucine is implicated in the haemoglobin production and the capacity to transport oxygen by RBC [83] and phenylalanine and tyrosine, play an important role in the synthesis of dopamine and norepinephrine neurotransmitters [84]. However, further studies are mandatory to assess the correlation of the metabolite levels with specific clinical features or comorbidities.

Spearman’s correlations and PLS regression revealed that many of the metabolic changes between controls and T2D patients could be correlated with Hb1Ac and plasmatic glucose levels. This result is coherent with the assumption that the main metabolic pathways in RBCs are affected by T2D, including the Embden-Meyerhof pathway, the PPP pathway and the glutathione pathway. In addition, the high correlation between the essential amino acids content versus HbA1c and plasmatic glucose was especially interesting, as these metabolites are not associated to any metabolic pathway in RBCs, and may be candidates to use as a complement of HbA1c and avoid its limitations. 

Interestingly, PLS and Spearman correlations agreed that some metabolites correlated mainly with HbA1c (creatine, ATP and GSH) while others correlated more with plasmatic glucose (6-phosphogluconate and valine). This means that RBCs metabolomics could not only help to improve HbA1c based T2D diagnosis and management, but also provide information about glycaemic variability. 

In an attempt to mimic the situation of hyperglycaemia in diabetes with an in vitro model, we were able to reproduce part of the changes observed in patients (Table 2). For instance, we detected an increase in lactate and 6-phosphogluconate in cultured RBCs, confirming that high glucose levels induced an alteration of the PPP and the Embden-Meyerhof pathway in vitro. However, changes on glutathione metabolism were much less pronounced. This result agree with previous studies showing that RBCs grown with higher glucose levels had a longer supply of energy source preventing the loss of GSH [42]. 

The current study shows some concerning limitations of the study. Thus, it is worth mentioning that despite the robustness of the results corroborated by an independent validation cohort, the total number of samples was small. Therefore, future studies should include a larger cohorts in multi-centre trial to confirm our results, and test their general applicability. In addition, it would be interesting to evaluate the usefulness of RBCs metabolic profile in predicting the conversion of metabolic syndrome in T2D. Furthermore, in this pilot study, T2D cases with relatively good glycaemic control were chosen, and therefore, confirmation of our results in T2D population with worse glycaemic control is needed. Another limitation of the study in terms of clinical applicability is the lack in most hospitals of high-resolution NMR spectrometers. In any case, NMR would be an optimal technique to detect this type of compounds. 

## 5. Conclusions 

In summary, we present for the first time a proof of concept showing how the metabolic profile of RBCs is altered in T2D. Results revealed significant differences that were confirmed with a small validation cohort. Some metabolites were related with alterations in the Embden-Meyerhof pathway, the Luebering-Rapoport pathway, the PPP and GSH synthesis, while others reflected an imbalance in metabolites transported by RBCs, mainly essential amino acids. Metabolic changes could be correlated with HbA1c and plasmatic glucose levels, confirming their relevance in T2D. Metabolic changes unrelated to metabolic pathways of RBCs, seem of great interest to use alone or in combination with HbA1c for identifying patients at risk of comorbidities. In addition, several metabolic changes detected in this pilot study were reproduced by a translational in vitro model. Further studies with larger cohorts are required to prove the consistency of these biomarkers, and if they could be related to specific comorbidities.

## Figures and Tables

**Table 1:** Main biochemical and anthropometrical clinical features of the three cohorts studied indicated as mean ± SEM.

	CT	CT Validation	T2D	*p* * CT/T2D	T2D Validation	*p* CT/T2D Validation
	n = 21	n = 6	n = 22		n = 12	
Age	60.9 ± 1.2	59.0 ± 3.4	64.3 ± 1.2	0.240	62.6 ± 2.2	0.700
Gender (female/male)	10/11	3/3	11/11		6/6	
HbA1c	5.6 ± 0.1	5.8 ± 0.2	7.0 ± 0.1	<0.001	7.3 ± 0.3	<0.001
BMI	34.9 ± 1.8	27.5 ± 1.9	31.8 ± 0.9	0.408	31.4 ± 1.1	0.424
HOMA	3.1 ± 0.5	2.3 ± 0.5	7.6 ± 1.4	0.029	8.7 ± 1.7	0.004
Chol (mg/dL)	216.8 ± 10.6	248.0 ± 15.4	170.7 ± 9.4	0.016	175.3 ± 8.6	0.081
HDL (mg/dL)	60.0 ± 2.9	58.6 ± 5.0	43.9 ± 1.8	<0.001	48.1 ± 2.9	0.081
LDL (mg/dL)	131.0 ± 9.1	170.2 ± 14.6	91.6 ± 5.7	0.005	103.5 ± 5.5	0.185
TG (mg/dL)	130.4 ± 8.8	141.8 ± 30.2	139.4 ± 13.9	0.832	117.8 ± 15.6	0.700
Glucose (mg/dL)	92.8 ± 3.5	97.7 ± 5.4	94.0 ± 7.9	0.893	134.8 ± 10.8	0.001
Insulin	13.5 ± 1.9	9.3 ± 1.7	17.9 ± 3.3	0.595	23.5 ± 6.3	0.258

* Statistical significance was determined using the Holm-Sidak method, with alpha = 0.05. Each row was analysed individually, without assuming a consistent standard deviation (SD). Healthy control individuals (CT), healthy control validation individuals (CT validation), T2D patients (T2D), and T2D patients for validation (T2D validation), glycosylated haemoglobin (HbA1c), body mass index (BMI), homeostatic model assessment (HOMA), cholesterol (Chol), high-density lipoprotein (HDL), low-density lipoprotein (LDL) and triglycerides (TG).

**Table 2:** Misclassification table for control (CT) and type 2 diabetes mellitus (T2D) prediction.

	Members	Correct	T2D	CT	No Class (YPred <= 0)
T2D	22	100%	22	0	0
CT	21	95.24%	1	20	0
No class	18		11	7	0
Total	61	97.67%	34	27	0
Fisher’s prob.	2.1 × 10^−11^				

Prediction results for the CT and T2D validation group with the orthogonal partial least squares discriminant analysis (OPLS-DA) model from CT/T2D.

**Table 3:** Significant metabolites in partial least squares (PLS) discriminant analysis versus glycosylated haemoglobin (HbA1c).

Metabolite	Bucket (ppm)		Loading	VIP
**2,3-Bisphosphoglycerate**	4.030	4.070	0.48	4.12
creatine	3.035	3.045	0.24	2.52
ATP	3.880	3.920	0.01	1.79
glucose	4.190	4.327	0.21	1.78
6_phosphogluconate	4.080	4.098	0.03	1.57
lactate	4.098	4.146	−0.13	1.33
GSH	1.472	1.500	0.10	1.19
alanine	4.563	4.580	−0.13	1.08
glutamine	2.380	2.410	−0.13	1.09
pyroglutamate	2.430	2.49	−0.12	1.08
glycine	3.553	3.575	0.11	1.03
aspartate	2.790	2.840	−0.09	0.94

Metabolites with significant variable importance for projection (VIP) values. Loadings of the first component are indicated.

**Table 4:** Significant metabolites partial least squares (PLS) discriminant analysis versus glucose.

Metabolite	Bucket (ppm)		Loading	VIP
2,3-Bisphosphoglycerate	4.030	4.070	0.49	4.16
6_phosphogluconate	4.080	4.098	−0.23	2.15
glucose	3.88	3.92	0.16	1.90
lactate	4.098	4.146	−0.12	1.75
pyroglutamate	2.380	2.410	−0.19	1.68
ATP	4.190	4.327	0.14	1.21
GSSG	2.430	2.490	−0.12	1.12
glutamine	2.790	2.840	−0.14	1.13
aspartate	3.290	3.333	0.13	1.17
succinate	2.400	2.410	0.09	1.06
glycine	3.553	3.575	0.09	0.83
GSH	1.472	1.500	0.02	0.84
alanine	4.563	4.580	−0.11	0.85

Metabolites with significant variable importance for projection (VIP) values. Loadings of the first component are indicated.

**Table 5:** Spearman correlations of selected significant metabolites against glucose and glycosylated haemoglobin (Hb1Ac).

	Glucose		HbA1c	
	rho	*p*	rho	*p*
2,3-Bisphosphoglycerate	0.2135	0.1366	0.4919	**0.0003**
6-phosphogluconate	−0.4385	**0.0014**	−0.3383	**0.0163**
aspartate	−0.3911	**0.0050**	−0.3376	**0.0165**
ATP	0.2795	0.0514	0.4606	**0.0008**
creatine	0.1571	0.2761	0.5522	**<.0001**
glucose	0.3642	**0.0119**	0.4149	**0.0027**
glycine	0.1942	0.1765	0.3131	**0.0269**
GSH	−0.3629	**0.0096**	−0.5944	**<0.0001**
GSSG	0.1176	0.4159	0.1919	0.1818
IMP	−0.2611	0.0670	−0.5624	**<0.0001**
leucine	−0.3541	**0.0116**	−0.4369	**0.0015**
lysine	−0.3467	**0.0136**	−0.2765	**0.0497**
phenylalanine	−0.1145	0.4287	−0.4739	**0.0005**
tyrosine	−0.1449	0.3155	−0.4662	**0.0006**
valine	−0.3635	**0.0095**	−0.3287	**0.0198**

Control individuals and both cohorts of type 2 diabetes mellitus (T2D) patients were included. Significant *p*-values are labelled in bold.

**Table 6:** Glycosylated haemoglobin (Hb1A)c levels in cultured red blood cells (RBCs).

Time (h)	Hb1Ac (%) 4.5 g/L Glucose	Hb1Ac (%) 9.5 g/L Glucose	Hb1Ac (%) 20.0 g/L Glucose
0	3.98 ± 0.01	3.98 ± 0.01	3.98 ± 0.01
24	5.81 ± 0.54	6.01 ± 0.60	6.71 ± 0.75

RBCs were incubated with different concentration of glucose at 0 and 24 h.

**Table 7:** Altered metabolites found on incubated red blood cells (RBCs).

Metabolite	4.5 g/L Glucose	20 g/L Glucose	*p* (Student)	Comparison In Vivo/In Vitro
glucose	22.638 ± 0.607	240.987 ± 13.421	**0.0002**	**Similar changes in vivo and in vitro**
6-phosphogluconate	4.829 ± 0.145	3.923 ± 0.082	**0.0136**	
glutamate	14.463 ± 0.194	17.138 ± 0.081	**0.0006**	
succinate	6.224 ± 0.250	7.157 ± 0.073	**0.0500**	
lactate	243.810 ± 10.070	168.783 ± 2.033	**0.0048**	
lysine	5.211 ± 0.066	6.289 ± 0.037	**0.0004**	**opposite changes in vivo and in vitro**
glutamine	16.882 ± 0.319	19.100 ± 0.278	**0.0154**	
pyroglutamate	12.181 ± 0.285	13.990 ± 0.119	**0.0105**	
valine	31.432 ± 2.171	65.278 ± 0.857	**0.0004**	
ATP	5.324 ± 0.394	2.348 ± 0.162	**0.0056**	
alanine	13.825 ± 0.470	11.984 ± 0.075	**0.0401**	
acetate	4.552 ± 0.043	5.859 ± 0.033	**0.0000**	**significant changes only in vitro**
malate	1.784 ± 0.044	2.026 ± 0.018	**0.0172**	
phosphocholine	5.383 ± 0.145	4.662 ± 0.111	**0.0375**	
glutamine	16.882 ± 0.319	19.100 ± 0.278	**0.0154**	
pyroglutamate	12.181 ± 0.285	13.990 ± 0.119	**0.0105**	

RBCs were incubated at 0 and 24 h with 4.5 and 20 g/L of glucose. Relative normalised intensities are indicated as mean ± SEM. ATP = adenosine triphosphate.

## Supplementary Materials

The following are available online at https://www.mdpi.com/2077-0383/9/6/1619/s1, Figure S1: PCA score plots of metabolomic profiles of RBCs of patients included in the study. Figure S2: OPLS-DA analysis of the metabolomic profile of RBCs of all samples included in the study. Table S1: Identified polar metabolite signals in 1H NMR spectra. Table S2: Normalised concentration values of metabolites in RBCs of T2D patients versus controls.
