# Supplementary material for: A Translational In Vivo and In Vitro Metabolomic Study Reveals Altered Metabolic Pathways in Red Blood Cells of Type 2 Diabetes
Source: J Clin Med. 2020 May 27;9(6):1619. doi: 10.3390/jcm9061619 (PMC7355709; doi:10.3390/jcm9061619)
Supplement: Supplementary file 1 [file jcm-09-01619-s001.pdf]

Supplementary Materials

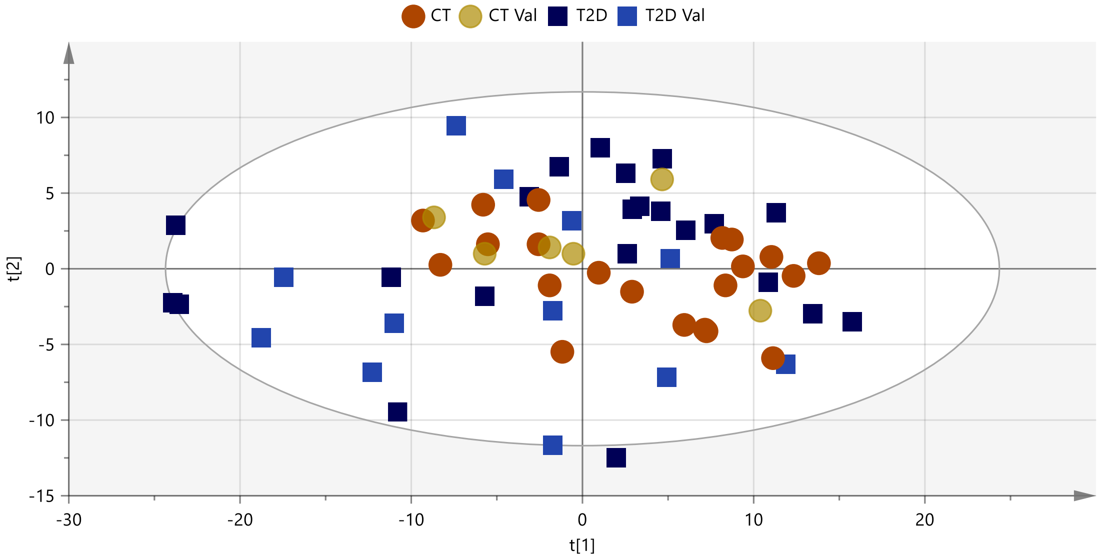

Figure S1. PCA score plots of metabolomic profiles of RBCs of patients that participated in the study. Data were UV scaled and mean centred. R2X(cum) = 0,837, Q2(cum) = 0.665.

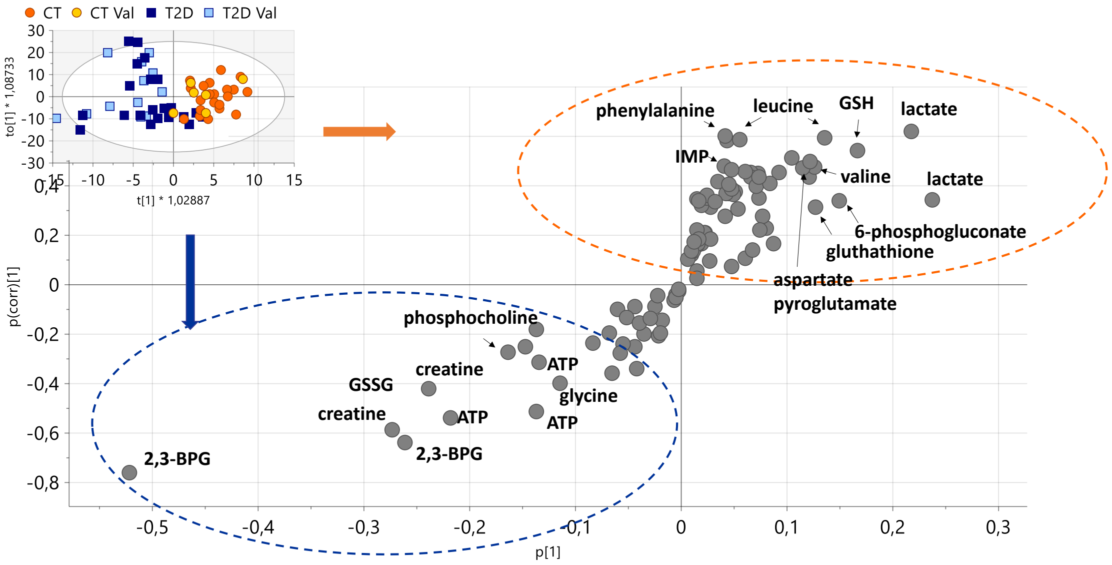

Figure S2. R2Y(cum)=0,606 Q2(cum)=0,532 Permutation: R2 = (0.000, 0.132), Q2 = (0.000, -0,247). ANalysis Of VAriance testing of Cross-Validated predictive residuals (CV-Anova): p-value=9.3e9. IMP = inosine monophosphate, ATP = adenosine triphosphate, GSH, = reduced glutathione, GSSG = oxidized glutathione, 2,3-BPG = 2,3-Bisphosphoglyceric acid. Data were pareto scaled and mean centred.

Table S1. Identified metabolite signals in ^{1}H NMR spectra of polar extracts with integration regions in ppm, multiplicity, TOCSY and HSQC correlation peaks. S = singlet, d = doublet, dd = double doublet, t = triplet, m = multiplitet, n/v = not visible.

Metabolite	left limit	right limit	multiplicity	TOCSY correlation peaks	HSQC correlation peak
**2,3_diphosphoglycerate**	4,070	4,030	m	4.52	66.4
**2,3_diphosphoglycerate**	4,503	4,563	m	4.05	75.0
**2-aminoisobutyrate**	1,460	1,470	s	-	26.3
**2-hydroxybutyrate**	0,860	0,911	t	1.64	11.3
**4_pyridoxate**	7,839	7,855	s	-	137.3
**6_phosphogluconate**	3,830	3,840	m	overlapped	70.9
**6_phosphogluconate**	4,080	4,098	m	overlapped	70.6
**6_phosphogluconate**	4,170	4,190	m	overlapped	73.7
**acetate**	1,910	1,928	s	-	26.11
**ADP**	8,340	8,369	s	-	149.6
**alanine**	1,470	1,500	d	3.80	19.0
**AMP**	4,490	4,503	m	4.37	73.4
**aspartate**	2,790	2,840	dd	2.67	36.7
**aspartate**	2,660	2,685	dd	2.81	36.7
**ATP**	4,030	3,979	m	overlapped	66.4
**ATP**	4,190	4,327	m	overlapped	65.0
**ATP**	4,595	4,634	t	overlapped	70.0
**ATP**	8,257	8,295	s	-	152.8
**ATP+ADP**	6,136	6,179	d	4.60	86.8
**ATP+ADP**	8,507	8,519	s	-	140.0
**ATP+AMP**	4,351	4,440	m	overlapped	84.0
**betaine**	3,191	3,214	s	-	55.7
**creatine/phoshocreatine**	3,930	3,950	s	-	39.8
**creatine/phosphocreatine**	3,035	3,045	s	-	57.5
**CTP/CDP**	7,925	7,947	d	-	144.2
**ethanol**	1,170	1,200	t	3.65	19.8
**formate**	8,454	8,469	s	-	172.4
**GDP/GTP**	5,938	5,963	d	4.72	89.4
**glucose**	4,640	4,670	d	3.52	98.7
**glucose**	5,232	5,248	d	3.52	94.9
**glutamate**	2,330	2,380	m	2.08	36.3
**glutamate**	2,030	2,100	m	2.33	30.0
**glutamate + glutamine**	2,100	2,140	m	2.33 (glutamate) 2.43 (glutamine)	30.0 (glutamate) 29.3 (glutamine)

**glutamine**	2,430	2,490	q	2.12	33.9
**glycerol**	3,570	3,590	dd	3.65	62.1
**glycerol**	3,635	3,690	dd	3.58	62.1
**glycine**	3,553	3,575	s	-	56.4
**glycolate**	3,844	3,860	s	-	60.7
**GPC+carnitine**	3,205	3,214	s	-	56.4
**GSH**	2,879	2,901	m	2.54	41.6
**GSH**	4,563	4,580	dd	4.58	56.5
**GSH + GSSG**	2,140	2,220	m	2.54	29.2
**GSH+GSSG**	2,500	2,600	m	2.17	34.3
**GSSG**	2,950	3,010	m	overlapped	39.4
**GSSG**	3,290	3,330	m	overlapped	39.4
**guanidino+guanido acetate**	3,760	3,800	s	-	44.0
**guanosine**	7,750	7,760	s	-	104.6
**hidroquinone**	6,800	6,811	s	Confirmed with spiking	
**histidine**	8,115	8,124	s	Confirmed with spiking	
**histidine+histamine**	7,074	7,095	s	Confirmed with spiking	
**homocysteine**	2,620	2,635		-	19.4
**hypoxanthine**	7,916	7,924	s	Confirmed with spiking	
**isoleucine**	1,007	1,027	d	1.27	17.62
**lactate**	1,320	1,350	d	4.10	22.5
**lactate**	4,098	4,146	q	1.32	71.2
**leucine**	0,983	0,951	t	1.72	23.9<br>24.8
**lysine**	1,505	1,526	m	1.90	23.9
**lysine**	1,526	1,532	m	1.90	23.9
**lysine**	1,870	1,900	m	1.52	32.8
**lysine**	1,928	1,940	m	1.52	32.8
**malate**	2,712	2,726	dd	-	43.0
**malonate**	3,090	3,110	s	Confirmed with spiking	
**methanol**	3,350	3,387	s	-	51.8
**methionine**	2,645	2,660	m	n/v	28.6
**NAD**	8,840	8,854	d	n/v	148.4
**NAD**	9,137	9,161	d	n/v	145.2
**NAD**	9,335	9,352	s	n/v	142.8
**NADH/NADPH**	6,930	6,960	s	-	1391
**NADP**	8,825	8,840	d	n/v	148.4
**NADP**	9,292	9,308	s	n/v	145.2
**nicotinamide**	8,140	8,160	s	-	136.7
**PC**	3,190	3,205	s	-	56.4

**phenylalanine**	7,320	7,330	m		130.5/132.1/ 132.5
**phosphoenolpiruvate**	5,363	5,380	s	Confirmed with spiking	
**phosphoethanolamine**	3,979	3,984	m	overlapped	60.4
**proline**	3,390	3,400	m	2.01	48.8
**proline**	4,147	4,151	m	2.01	63.9
**pyridoxamine**	7,678	7,695	s	-	123.8
**pyroglutamate**	2,380	2,410	m	Confirmed with spiking	
**pyruvate**	2,340	2,360	s	Confirmed with spiking	
**spermidine/spermine**	3,110	3,150	m	overlapped	44.3
**succinate**	2,400	2,410	s	-	36.3
**taurine**	3,400	3,450	t	3.29	39.0
**thymidine**	6,320	6,360	s	Confirmed with spiking	
**tryptophane**	7,530	7,540	d		111.5
**tyrosine**	6,891	6,922	d	6.91	118.4
**tyrosine**	7,186	7,216	d	7.20	133.7
**UDP derivatives/NADH/NADPH/CTP**	5,960	6,010	m		
**UDP derivatives/NADH/NADPH/CTP**	6,031	6,058	m		
**UDP-glucose + UDP-galactose**	5,589	5,620	m	7.96	98.2
**UDP-glucose+CTP**	7,947	7,975	m	5.98	144.2
**UDP-NAG**	5,446	5,472	m	5.99	97.4
**uracil+tryptophane**	7,540	7,554	d	-	111.5 (tryptophan) 143.0 (uracil)
**valine**	0,984	1,006	d	2.24	19.67
**valine**	1,035	1,061	d	2.24	20.84
**valine**	2,240	2,310	m	1.0/1.05	32.01
**xanthine+guanine**	7,554	7,559			140.5 (xanthine) 149.0 (guanine)

Table S2. Relative normalized intensities values found in RBCs of T2D patients with control of the pathology against controls. Values are indicated as are indicated as mean±SEM* Statistical significance was determined using the Holm-Sidak method, with alpha = 0.05. Each row was analyzed individually, without assuming a consistent standard deviation (SD).

metabolite	right limit (ppm)	left limit (ppm)	CT	CT val	T2D	T2D val	p* CT/T2D	p CT/T2D val	CT+CT val	T2D +T2D val	p CT+CT val/T2D+T2D val
2,3-Bisphosphoglycerate	4,503	4,563	17,0±0,6	15,7±1,2	20,3±1,0	22,7±1,6	0,0778	0,0051	16,7±0,6	0,0051	21,2±0,9
6-phosphogluconate	4,080	4,098	19,8±0,6	14,1±3	18,0±1,1	10,8±0,6	0,4809	<0,000001	18,6±0,9	<0,000001	15,4±0,9
alanine	1,472	1,500	13,7±0,4	15,5±2	16,5±0,8	15,3±1,2	0,0433	0,2851	14,1±0,5	0,2851	16±0,7
aspartate	2,790	2,840	8,4±0,3	7,2±0,7	7,6±0,6	6,2±0,5	0,5474	0,0049	8,2±0,3	0,0049	7,1±0,4
ATP	4,190	4,327	33,7±0,8	35,8±1,2	37,1±1,1	40,7±0,7	0,1065	0,0000	34,1±0,7	0,0000	38,4±0,8
creatine	3,930	3,950	17,6±0,6	20,9±1,8	23,5±1,7	23,3±1,2	0,0418	0,0004	18,4±0,6	0,0004	23,4±1,2
glucose	5.232	5.248	1.7±0.2	**4,2±1,4**	0.5±0.1	1.5±0.4	1.54E-06	0.7746	2,2±0,4	0.7746	0,9±0,2
glutamate	2,330	2,380	11,8±0,3	14±1,2	12,4±0,5	13,0±0,6	0,5492	0,2851	12,3±0,4	0,2851	12,6±0,4
glycine	3,553	3,575	9,4±0,3	10,5±0,8	11,2±0,6	11,0±0,7	0,1001	0,1885	9,7±0,3	0,1885	11,2±0,4
GSH	4,563	4,580	5,2±0,2	2,7±0,7	3,7±0,5	2,2±0,2	0,0497	<0,000001	4,7±0,3	<0,000001	3,1±0,4
GSSG	3,290	3,333	13,3±0,4	17,8±1,1	15,6±0,6	15,6±1,5	0,0682	0,2851	14,3±0,5	0,2851	15,6±0,7
IMP	8,230	8,240	0,6±0,1	0,3±0,1	0,4±0,0	0,4±0,0	0,0337	0,0422	0,5±0	0,0422	0,4±0
lactate	4,098	4,146	19,2±0,6	16,6±0,6	16,2±0,7	14,8±1,2	0,0255	0,0084	18,6±0,5	0,0084	15,7±0,6
leucine	0,951	0,983	7,4±0,2	8,4±0,6	6,2±0,3	5,8±0,3	0,0337	0,0017	7,6±0,2	0,0017	6±0,2
lysine	1,526	1,532	0,8±0,0	0,8±0,1	0,8±0,1	0,6±0,1	0,5492	0,0446	0,8±0	0,0446	0,7±0
phenylalanine	7,322	7,335	0,7±0,0	0,7±0,1	0,5±0,0	0,6±0,0	0,0085	0,2561	0,7±0	0,2561	0,6±0
phosphocholine	3,190	3,205	4,7±0,2	3,8±0,4	5,5±0,4	4,4±0,5	0,2672	0,5272	4,5±0,2	0,5272	5,1±0,3
phosphocreatine	3,045	3,052	3,2±0,1	3,8±0,2	3,8±0,2	3,6±0,3	0,1461	0,2851	3,3±0,1	0,2851	3,7±0,2
tyrosine	7,186	7,216	1,9±0,1	1,4±0,2	1,5±0,1	1,6±0,1	0,0353	0,1077	1,8±0,1	0,1077	1,5±0,1
valine	1,035	1,061	6,0±0,2	6,6±0,4	5,2±0,3	4,8±0,2	0,1289	0,0084	6,1±0,2	0,0084	5±0,2
